# Late-replicating CNVs as a source of new genes

**DOI:** 10.1242/bio.20136924

**Published:** 2013-11-14

**Authors:** David Juan, Daniel Rico, Tomas Marques-Bonet, Óscar Fernández-Capetillo, Alfonso Valencia

**Affiliations:** 1 Structural Biology and BioComputing Programme, Spanish National Cancer Research Center (CNIO), Melchor Fernández Almagro 3, 28029 Madrid, Spain; 2 Institut Catala de Recerca i Estudis Avancats (ICREA) and Institut de Biologia Evolutiva (UPF/CSIC), Dr Aiguader 88, PRBB, 08003 Barcelona, Spain

**Keywords:** CNV, DNA replication timing, Duplicated genes, Evolution

## Abstract

Asynchronous replication of the genome has been associated with different rates of point mutation and copy number variation (CNV) in human populations. Here, our aim was to investigate whether the bias in the generation of CNV that is associated with DNA replication timing might have conditioned the birth of new protein-coding genes during evolution. We show that genes that were duplicated during primate evolution are more commonly found among the human genes located in late-replicating CNV regions. We traced the relationship between replication timing and the evolutionary age of duplicated genes. Strikingly, we found that there is a significant enrichment of evolutionary younger duplicates in late-replicating regions of the human and mouse genome. Indeed, the presence of duplicates in late-replicating regions gradually decreases as the evolutionary time since duplication extends. Our results suggest that the accumulation of recent duplications in late-replicating CNV regions is an active process influencing genome evolution.

## Introduction

Not all genes in a genome accumulate mutations and evolve at the same rate (Wolfe et al., 1989; Stern and Orgogozo, 2009), a phenomenon for which diverse adaptive and non-adaptive mechanisms have been proposed (Stern and Orgogozo, 2009; Lynch, 2007; Demuth and Hahn, 2009). Recent studies suggest that replication timing (RT) during S-phase may be a non-adaptive factor that contributes to the bias in the accumulation of point mutations (Stamatoyannopoulos et al., 2009; Herrick, 2011; Koren et al., 2012). Indeed DNA replication errors constitute a major source of mutations, which represent the raw material for the evolution of the genome.

The dynamics of replication seems to be largely driven by the configuration of chromatin within the nucleus, whereby more open, physically connected chromosome territories rich in transcriptionally active genes replicate earlier than more tightly packed ones (Hansen et al., 2010; Yaffe et al., 2010; Ryba et al., 2010; De and Michor, 2011). We also know that asynchronous replication of eukaryotic genomes reflects the physical limitations that chromatin compaction exerts on DNA transactions (Ding and MacAlpine, 2011). Late replication of heterochromatic regions of the genome provokes the accumulation of single-stranded DNA (ssDNA), due to the difficulties experienced by DNA polymerase to fill in the gaps. Given that ssDNA is the substrate for recombination reactions that can alter the genome, the accumulation of ssDNA is known as “replication stress” (López-Contreras and Fernandez-Capetillo, 2010). Interestingly, evolutionary divergence and single-nucleotide polymorphisms (SNPs) tend to accumulate in late-replicating regions of the human genome, suggesting that during evolution, mutations might have arisen primarily as a consequence of replicative stress (Stamatoyannopoulos et al., 2009). The association between late replication and greater sequence divergence seems to be a general feature of eukaryote genomes and indeed, it has also been reported in the mouse (Pink and Hurst, 2010), yeast (Lang and Murray, 2011) and in flies (Weber et al., 2012).

Whereas point mutations might shape the function of existing genes, the birth of novel genes generally requires mechanisms that generate new genomic regions. Structural changes, such as copy number variants (CNVs), represent one of the main sources of intra- and inter-specific nucleotide differences between individuals (Zhang et al., 2009; Hastings et al., 2009; Mefford and Eichler, 2009). CNVs typically involve intermediate to large regions, providing a substrate for the generation of new genes through gene duplication. Pioneering studies detected pericentromeric and subtelomeric regions as hotspots of segmental duplications and CNVs (Bailey et al., 2001; Mefford and Trask, 2002; Nguyen et al., 2006; Bailey and Eichler, 2006). These regions were clearly enriched in recently expanded gene families, as well as in many repetitive non-coding elements (Horvath et al., 2001). Although other alternative mechanisms have also been proposed (Kaessmann, 2010), copy number variation is thought to be a major source of new genes (Kim et al., 2007; Korbel et al., 2008; Schuster-Böckler et al., 2010).

CNV formation in ancestral species might have led to genomic amplification of regions that contain genes. Later fixation of these regions in the population may occur when a percentage of individuals in a given species harbor a genomic region with an extra gene copy. Although further deletion or pseudogenization might often prevent such genes from becoming fixed (Zhang, 2003; Innan and Kondrashov, 2010), the accumulation of functional genetic changes can eventually lead to the establishment of new genes. An important effect of gene duplication is that evolutionary pressure can be shared between both duplicates due to their initial functional redundancy (Lynch and Force, 2000; Lynch et al., 2001; Zhang, 2003; Innan and Kondrashov, 2010). As a consequence, the duplication event not only creates a new copy of a given gene but also, it may modify the potential mutability of the parental copy, thereby facilitating the exploration of new functional solutions (Ross et al., 2013; Abascal et al., 2013). Interestingly, a significant fraction of the single nucleotide mutations accumulated during genome evolution can be the by-product of the DNA repair low-fidelity mechanisms involved in structural alterations, suggesting a close relationship between point mutations and genomic rearrangements (De and Babu, 2010).

Mechanistically, the models currently used to explain CNV formation involve either non-allelic homologous recombination (NAHR) of (macro or micro) homologous tracks, or non-homologous (NH) repair mechanisms that are at play during replicative stress (e.g. *Fork stalling and template switching* (FoSTeS) or *Microhomology-mediated break-induced replication* (MMBIR) (Hastings et al., 2009)). In humans, CNVs related with NH repair mechanisms are more frequently found in late-replicating regions, while NAHR CNVs tend to occur in early replicating regions (Koren et al., 2012). A relationship between late RT and CNV hotspots has also been reported in flies (Cardoso-Moreira et al., 2011). Furthermore, recent data suggest that somatic CNVs in cancer arise as a consequence of replicative stress (Dereli-Öz et al., 2011), and that chromosome structure and RT can be used to predict landscapes of copy number alterations in cancer genomes (De and Michor, 2011). Significantly, chemicals that promote replicative stress increase the rate of *de novo* CNV formation in human immortalized fibroblasts, strong evidence of a mechanistic role for replication stress in the generation of CNVs (Arlt et al., 2009; Arlt et al., 2011).

**Fig. 1.**
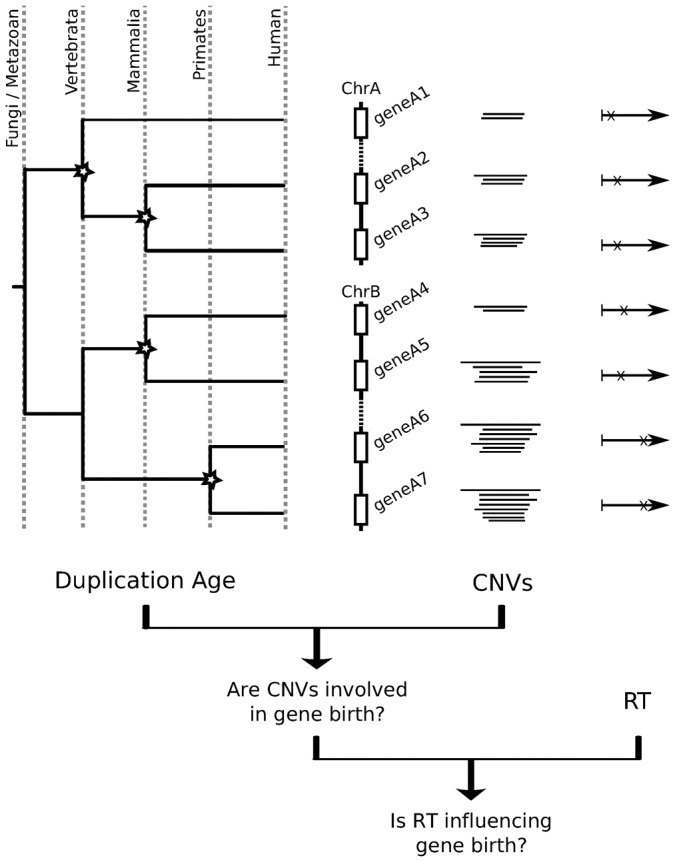
Summary of the analyses performed. This figure summarizes the analyses performed in this work, indicating the specific questions addressed and the datasets used. For each human protein-coding duplicated gene (PGD) we determined: (1) its duplication age, (2) whether it is within a CNV region in current human populations, and (3) its replication timing (RT) during S phase. We use this gene-centered information to investigate the involvement of CNVs in gene birth through duplication during human evolution and the possible influence of replication timing in these gene duplication events.

In this study we aimed to elucidate the possible relevance of the association of CNV regions with later DNA replication times on gene birth and evolution (a scheme representing the different elements analyzed is shown in Fig. 1). To address this key question, we followed an approach based on phylostratification, a framework that allows the evolutionary features of protein-coding genes to be identified and studied (Domazet-Lošo et al., 2007; Domazet-Lošo and Tautz, 2010; Roux and Robinson-Rechavi, 2011; Chen et al., 2012; Quint et al., 2012). Using this approach we found that RT and copy number variability in protein-coding duplicated genes (PDGs) are radically different depending on their evolutionary age. Our analyses also showed that most human genes duplicated in the Primate lineage are located in late-replicating CNV regions. Indeed, this relationship between recent gene duplication and late RT has probably been operating persistently and extensively throughout animal evolution, as we could see that RT parallels gene duplication age in different regions of the human and mouse genome. Our results suggest that molecular features of DNA transactions can influence current genomic structural variations, and that this influence has played a major role in the evolution of the mammalian genome. In particular, these events may facilitate the exploration of new functions through gene birth by duplication, leading to the characteristic distribution of protein function in mammalian genomes.

## Results

### CNV formation affects evolutionary recent PDGs

In this work, we studied the potential influence of DNA replication timing on the birth of new genes by duplication in the context of CNVs, recent duplication events that are not fixed but that are spread in populations. CNV regions are likely to be a source of future duplicated genes and evidence is accumulating that suggests their formation is associated to RT (Cardoso-Moreira et al., 2011; De and Michor, 2011; Koren et al., 2012). Therefore, we hypothesized that RT might be a relevant influence on the entire process of CNV generation and gene birth by duplication. Thus, we first examined the relationship between CNVs in human populations and gene duplication during metazoan evolution (Fig. 1).

**Fig. 2.**
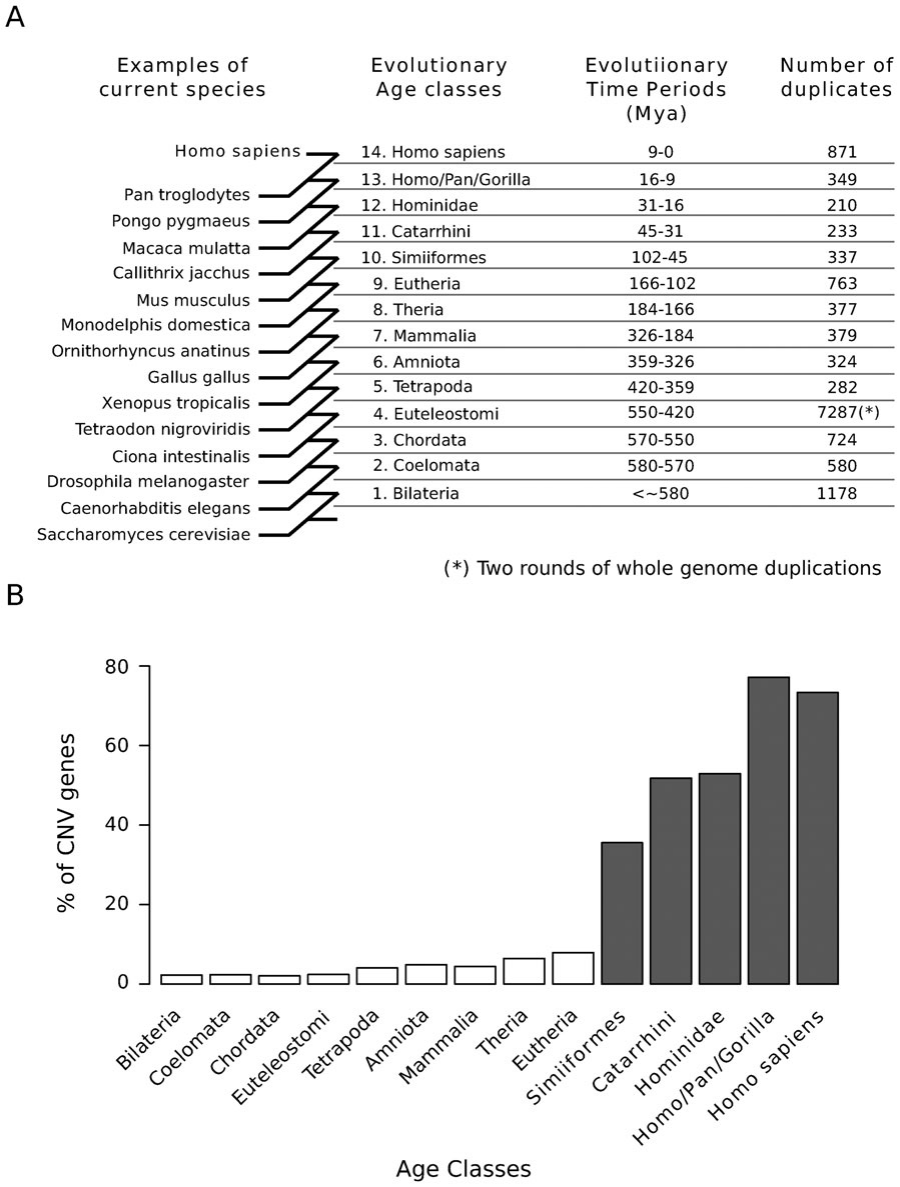
Phylostratification of human PDGs. (A) The age of a duplicated gene represents the ancestral species in which the duplication event that led to the generation of the extant gene was detected. A total of 13,909 gene duplicates were assigned to one of the 14 different evolutionary age groups (or phylostrata). Representative extant species that define the gene age classes are indicated (see Table 1 for the complete list). (B) The proportion of CNV genes in each phylostratum is higher in the genes recently duplicated in evolution (P-value <10^−150^, chi-squared test). A similar result was observed when only CNV gains are considered (supplementary material Fig. S1).

We quantified copy number variation of human protein-coding genes based on CNV maps for 153 humans genomes (Sudmant et al., 2010). Accordingly, we identified genes with CNVs (or CNV-genes) as the 1,092 autosomal protein-coding genes located in regions with either a gain or loss in at least two individuals (see Materials and Methods). We explored the association of gene CNV with duplication age (Fig. 1), which was established using a phylostratification protocol (Domazet-Lošo et al., 2007). As such, we assigned the evolutionary age of the last duplication in which it was involved to every human protein-coding duplicated gene (PDG) (Roux and Robinson-Rechavi, 2011; see Materials and Methods). Duplication events were dated according to 9,432 phylogenetic reconstructions of the 876,985 protein-coding genes from 51 metazoan species and *S. cerevisiae* (Flicek et al., 2011). In this way we were able to distinguish 5,339 protein-coding singleton genes (not duplicated since the appearance of the Metazoa) and 13,985 PDGs within this period of human evolution. Finally, we classified each PDG into 14 *age classes* or phylostrata corresponding to the ancestral species along the timeline of human evolution since the Fungi/Metazoa split (Fig. 2A; Table 1; also see Materials and Methods). This definition of evolutionary duplication age allows us to analyze the association of different genomic features with the age of the PDGs, helping us to understand the conditions of gene duplication.

**Table 1.**
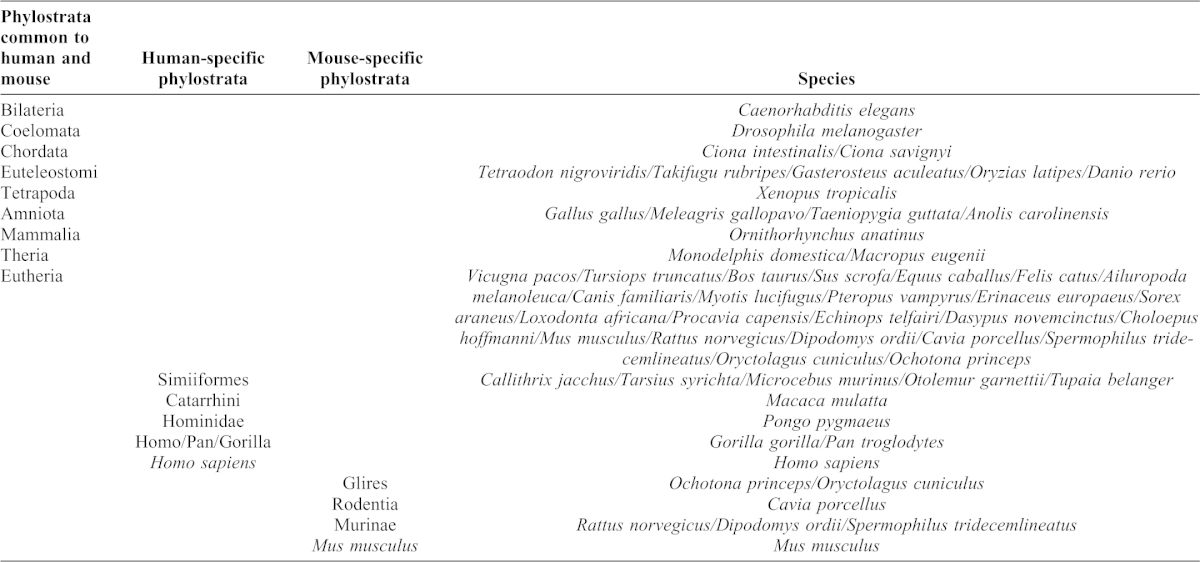
List of phylostrata used in the phylogenetic reconstructions.

We first observed that PDGs as a whole are more often found in human CNV regions than protein-coding singleton genes: 8% and 3%, respectively (P-value = 6.2×10^−31^). Having demonstrated a clear association between CNVs and gene duplication, we studied the distribution of CNV genes in different evolutionary duplication ages and we found that recent PDGs are clearly enriched in CNV-genes (Fig. 2B; P-value <10^−150^). Indeed, most PDGs duplicated since the primate ancestor were in CNV regions (61%), while most of the genes older than the Eutheria phylostratum (97%) seem to have completely fixed their copy number, which no longer varied in the human population (Fig. 2B).

These results imply that evolutionary recent PDGs are preferentially found in CNV regions, while genes that have not duplicated since the evolution of the first Primates (singletons and older PDGs) are rarely implicated in CNV formation.

### Evolutionary recent PDGs in CNV regions replicate later

The asynchronous DNA replication that occurs in the genome is related to different patterns of DNA damage, replicative stress and genome rearrangements (Stamatoyannopoulos et al., 2009; Yaffe et al., 2010; De and Michor, 2011; Koren et al., 2012). Most protein-coding gene-rich regions of the genome replicate early. This asymmetric distribution of genes in the genome might somehow reduce the deleterious effects associated to the higher mutation rate in late-replicating regions. In this scenario, we decided to investigate if the differences in RT between protein-coding genes were indeed associated to copy number variability.

**Fig. 3.**
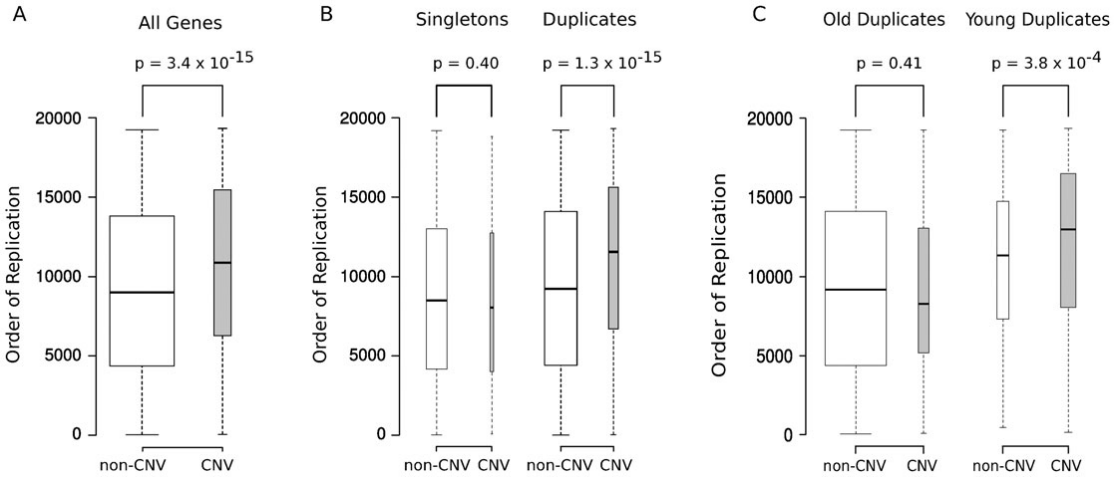
Gene duplications, CNVs and RT. (A) The box plots represent the RT of all human protein-coding genes. The RT was obtained from publicly available microarray-based RT maps. A total of 19,197 human genes were ranked from early to late according to their order of replication. Genes located in CNV regions (*CNV genes*) replicate later (P-value = 3.4×10^−15^, Wilcoxon's test). (B) PDGs in CNV regions replicate later than non-CNV PDGs (P-value = 1.3×10^−15^), a difference that was not observed for singleton genes (P-value = 0.40). (C) Young PDGs (genes duplicated in the primate phylostrata) are preferentially located in CNV regions that replicate late (P-value = 3.8×10^−4^, Wilcoxon's test), whereas the difference between CNV and non-CNV PDGs is not significant in older duplicates (P-value = 0.41). Note that PDGs duplicated during Primate evolution tend to replicate later than older genes (P-value = 3.9×10^−112^). The box width is proportional to the number of genes within each figure panel.

We calculated the RT of 19,197 human protein-coding genes using the genome-wide RT maps (Ryba et al., 2010) of four different human embryonic stem cell (ESC) lines, which represent the best proxy available for germ-line replication times (Pink and Hurst, 2010). For further analyses we used the *order of replication* of each gene from the genome-wide RT profiles, as a relative measure of the moment of replication of each human protein-coding gene (see Materials and Methods). Using these data, we found that CNV-genes replicated significantly later than non-CNV genes (Fig. 3A; P-value = 3.4×10^−15^). More interestingly, the association of CNV and late replication is distinct for singletons and PDGs. While CNV-PDGs replicate clearly later than non-CNV PDGs (Fig. 3B; P-value = 1.3×10^−15^), we did not observe such an association in singleton genes (Fig. 3B; P-value = 0.40).

Based on this observation, we wondered whether this association between CNV PDGs and RT would be even stronger for evolutionary recent genes. This possibility can be explored by differentiating between old and young PDGs (defined as those that duplicated before or after primates evolved). Indeed, we observed a very different behavior for these two age groups (Fig. 3C), whereby recently duplicated PDGs in CNV regions tend to replicate later than young non-CNV PDGs (Fig. 3C, P-value = 3.8×10^−4^), a trend that disappears completely for old PDGs (Fig. 3C, P-value = 0.41). These observations were compatible with a prevalent role of CNVs in gene birth through duplication during mammalian evolution. Furthermore, they support the existence of a strong association between recent protein-coding gene duplications and CNV formation in late-replicating regions.

### DNA replication timing reflects evolutionary age

**Fig. 4.**
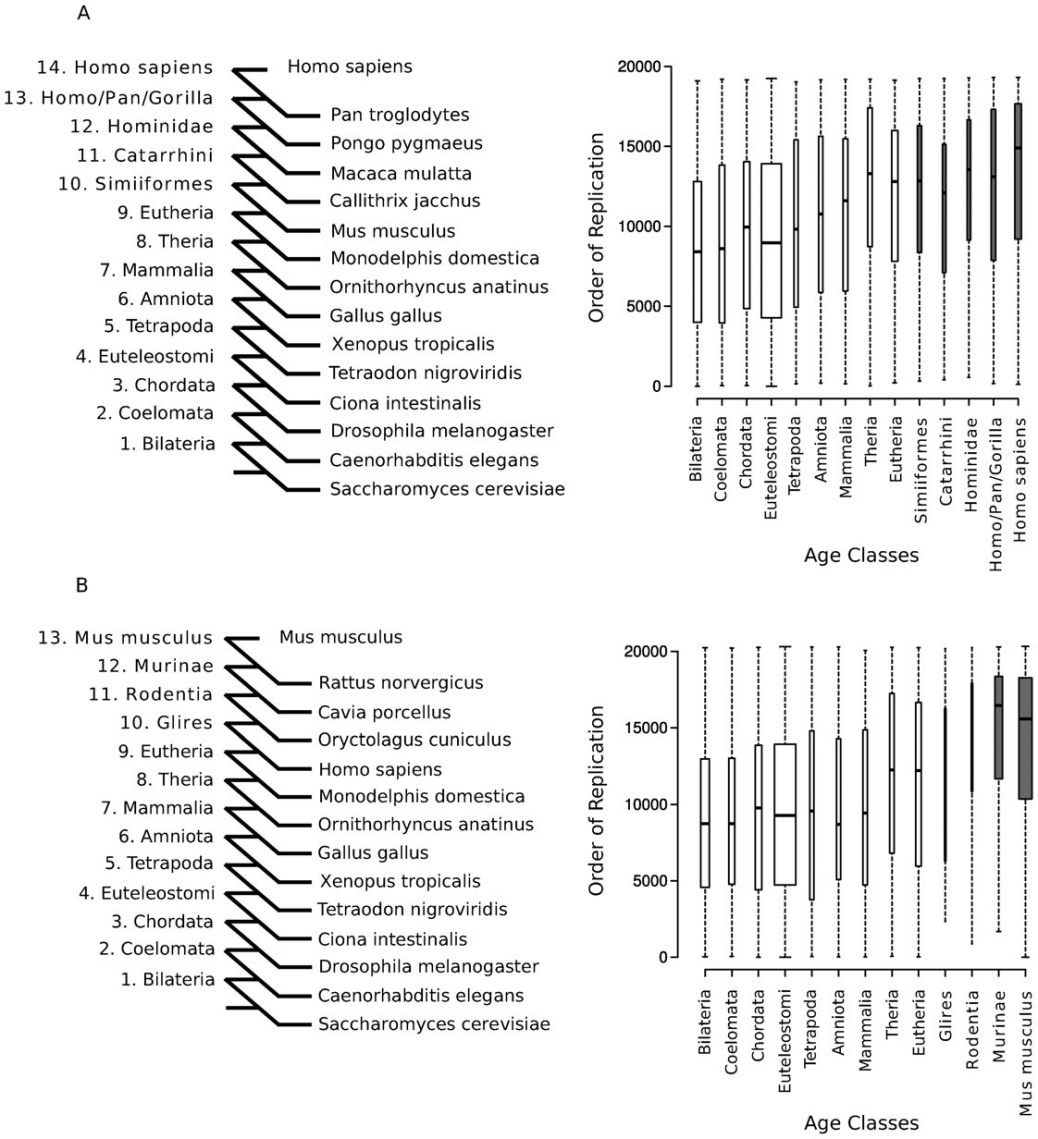
RT mirrors gene duplication phylogeny. (A) RT distribution of human PDGs is correlated with duplication age (rho = 0.21, P-value = 5.1×10^−150^, Spearman's correlation). (B) RT distribution of mouse PDGs is also correlated with duplication age (rho = 0.28, P-value = 5.8×10^−278^). The box width is proportional to the number of PDGs within each figure panel, and the specific human and mouse lineage age classes are indicated in bold. See also supplementary material Figs S2–S4.

It was evident from our previous analyses (Fig. 3C) that genes duplicated during primate evolution tend to replicate later than older genes (P-value = 3.9×10^−112^). Thus, we explored the association between gene duplication age and RT in detail, comparing RT in different phylostrata. Strikingly, we observed a clear correlation between RT and gene phylogeny, whereby younger genes gradually became more likely to be replicated later in the S phase (Fig. 4A; rho = 0.21, P-value = 5.1×10^−150^, Spearman's correlation). This trend is robust, even when we used the RT profiles of human lymphoblasts (Ryba et al., 2010) or fibroblasts (Yaffe et al., 2010) obtained using an alternative methodology (supplementary material Fig. S2).

To determine how widespread this correlation was in other mammals, we performed an independent analysis of 14,677 mouse PDGs using mouse ESC RT maps (Hiratani et al., 2010). Following the same phylostratification protocol used for human genes, we classified each mouse PDG according to the 13 age classes associated to the ancestral species in the evolutionary timeline of *Mus musculus* (Table 1; supplementary material Fig. S3). In this way, we again found that the younger mouse PDGs in the mouse genome tend to be late replicating (Fig. 4B; rho = 0.28, P-value = 5.8×10^−278^, Spearman's correlation). Therefore, the association of gene duplication age and RT appears to be highly significant in Primates and Rodents.

### DNA replication timing reflects evolutionary age at different chromosomal locations

Pericentromeric and subtelomeric regions have previously been described as hotspots of gene duplication (Mefford and Trask, 2002; Bailey and Eichler, 2006) and thus, we evaluated the contribution of these genomic regions to the trends observed in the previous section. We separated the human PDGs into three groups: pericentromeric (1,325 PDGs within 5 Mb from the centromere), subtelomeric (2,590 PDGs within 5 Mb from the telomere), and interstitial genes (the remaining 15,940 PDGs). Using the same definition, pericentromeric and subtelomeric regions in mouse contain many fewer PDGs (563 and 886, respectively), probably due to the fact that all the autosomal mouse chromosomes are acrocentric, with no protein-coding genes located in the short arms of the chromosome.

We found that PDGs duplicated in the specific human and mouse lineages are significantly enriched at pericentromeric regions of human (P-value = 5.1×10^−38^, chi-squared test) and mouse (P-value = 4.4×10^−5^) chromosomes. We did not observe a significant enrichment of PDGs duplicated during Primate or Rodent evolution in subtelomeric regions. However, both regions in human are enriched in CNV PDGs, with a 1.44 fold enrichment in subtelomeric regions (P-value = 4.6×10^−4^) and 2.35 fold enrichment in pericentromeric regions (P-value = 1.6×10^−19^). These observations are in agreement to previous estimates (Bailey et al., 2001) and suggest that the contribution of pericentromeric regions to the birth of new duplicates might have been particularly relevant during primates evolution.

**Fig. 5.**
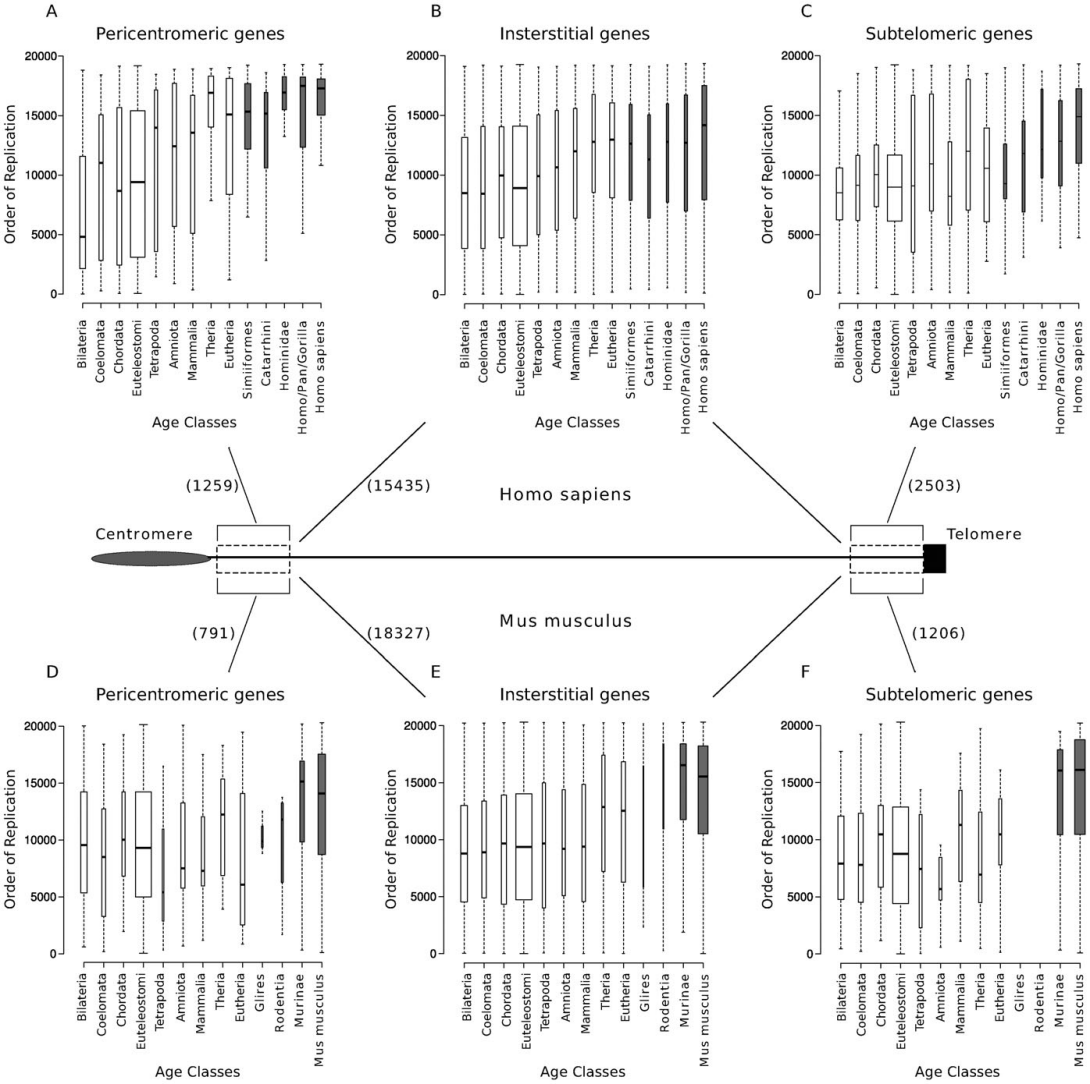
The association of PDG age and RT is observed in different human and mouse chromosomal regions. (A) Human pericentromeric regions (rho = 0.44, P-value = 1.1×10^−47^, Spearman's rank correlation). (B) Human interstitial regions (rho = 0.18, P-value = 2.7×10^−84^). (C) Human subtelomeric regions (rho = 0.23, P-value = 5.2×10^−24^). (D) Mouse pericentromeric regions (rho = 0.17, P-value = 2.0×10^−4^). (E) Mouse interstitial regions (rho = 0.29, P-value = 5.6×10^−255^). (F) Mouse subtelomeric regions (rho = 0.32, P-value = 3.6×10^−23^). Subtelomeric and pericentromeric PDGs were defined as those within 5 Mb of the telomere or centromere, respectively. The rest of the PDGs are considered to be in interstitial regions. The box width is proportional to the number of PDGs within each figure panel.

We next analyzed the RT of the PDGs in each of the three regions of human chromosomes. The correlation between gene RT and evolutionary age remains statistically significant when human pericentromeric, interstitial and subtelomeric PDGs are analyzed separately (Fig. 5A,B), although it was particularly strong for human pericentromeric PDGs (rho = 0.44, P-value = 1.1×10^−47^). We also performed a similar analysis for mouse genes and the association between gene age and RT was also significant for the three chromosomal regions (Fig. 5D–F). In the mouse, the general relationship between RT and gene age was stronger (rho = 0.29, P-value = 5.6×10^−255^) in interstitial regions, although it was also significant in the pericentromeric and subtelomeric regions. These observations highlight the prevalence of the association between RT and gene duplication, irrespective of the chromosomal regions where these evolutionary clades concentrate their gene birth events.

In conclusion, the younger the PDG is in evolutionary terms, the later it tends to replicate during S-phase in dividing cells. This surprising temporal parallel can be observed in different mammalian lineages and in different genomic regions. These data reinforce the view of RT as a fundamental element in the organization of the mammalian genome. Remarkably, this relationship can still be detected in the PDGs duplicated at different periods before the mammalian split (P-value = 0.02), suggesting that difficulties associated with late replication (such as replicative stress) might have exerted a strong influence on the evolution of new functions from the earliest stages in the evolution of multicellular organisms.

## Discussion

We have shown here that protein-coding genes duplicated in evolution (PDGs) are preferentially located in CNV regions. These CNV PDGs are prone to replicate later than non-CNV PDGs, suggesting a link between CNVs, gene duplication and late replication in human cells. We performed a precise phylostratification analysis to determine the ancestral species in which each human PDG was duplicated for the last time. PDGs duplicated after the common *Primate* ancestor were seen to be much more likely to be located in human CNV regions, suggesting that copy number variation in current populations and the fixation of new PDGs are two extremes of a continuous process.

We also observed that *Primate* CNV PDGs replicate even later than *Primate* non-CNV PDGs. This tendency was not observed for older PDGs, which tend to replicate early even if they are located in CNV regions. These results also suggest that copy number formation in gene coding regions is affected distinctly by two mechanisms recently associated to RT. Accordingly, early replicating CNVs are frequently linked to recombination mechanisms such as NAHR, while late-replicating CNVs are more frequently associated to non-homology (NH) based mechanisms (Koren et al., 2012) generally associated with replication errors (Hastings et al., 2009). Therefore, singletons and older duplicates that are associated with CNV events would generally be early replicating and involved in recombination events, while CNVs affecting young genes would tend to replicate late as a result of NH mechanisms.

Interestingly, we have also shown that RT mirrors the evolutionary age of PDGs in both human and mouse genomes, where younger PDGs tend to replicate later. Indeed, the replication of primate and rodent specific PDGs (protein-coding genes duplicated after the split from their common ancestor) is clearly enriched in the late S-phase. These observations suggest that there is an active process causing newborn duplicated genes to progressively accumulate in the late-replicating genomic regions. Although we propose that gene duplication associated to structural variations such as CNVs may be an important factor explaining this trend, retropositions have also been shown to be a source of gene duplicates (Kaessmann, 2010). Given that the trends we observed here are general for all detectable duplicates, future studies will be needed to address the possible differences between duplicates of different origin.

The regular trends observed at distinct evolutionary ages indicate that this process might have been in operation since ancient periods of metazoan evolution. Moreover, this association clearly persists when we analyze pericentromeric, interstitial and subtelomeric regions separately (regions differentially associated to structural variations (Mefford and Trask, 2002; Bailey and Eichler, 2006)). These results must be understood in the light of the recently defined “time-invariant principles” of genome evolution (De and Babu, 2010) that refer to aspects of genome evolution that are actually detected at very different time-scales (from cell lifetime to long evolutionary periods). In fact, the parallel between DNA replication and the evolution of gene families by duplication highlights the connection between two processes that occur over extremely different time scales. Eukaryotic DNA replication is completed over approximately 10 hours in dividing human cells, while gene phylogeny represents the accumulated process of gene birth (and loss) over hundreds of millions of years of evolution. In this context, our results indicate that structural and dynamic features of the genome could condition the evolution of its functional organization.

The robustness of the association between duplication age and RT led us to conceptually explore the possible implications of our results in the context of other recent discoveries. It is known that late-replicating regions are gene poor in general and particularly deployed of housekeeping/essential genes. In consequence, the insertion of the duplicated material on these regions is very unlikely to be problematic for the new cell. Therefore, the accumulation of new duplicates in these regions could actually facilitate the high rates of gene birth observed in complex species (Prince and Pickett, 2002). In addition, heterochromatin, also defined as *the chromatin that replicates late* (Beisel and Paro, 2011), is a structure clearly associated with late RT, and it can regulate cell type and tissue specific expression. Hence, the chromatin environment in which new genes arise might inherently restrict their expression, thereby reducing their impact on the whole organism while facilitating specific adaptations. This implies that the genomic context where new genes would contribute to the smaller selective pressures found in new genes (Albà and Castresana, 2005; Wolf et al., 2009; Vishnoi et al., 2010).

**Fig. 6.**
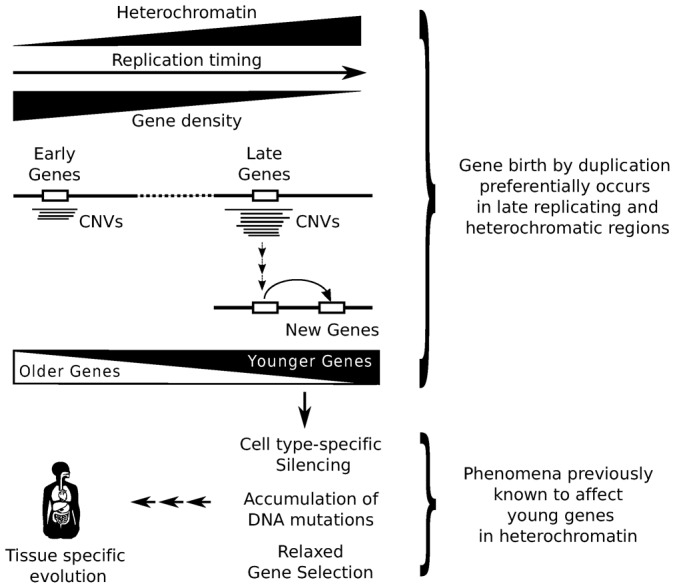
Proposed model based on our observations and previous knowledge. According to our results, a bias in CNV formation (probably associated with replicative stress) leads to the accumulation of CNV-genes in heterochromatin-rich, late-replicating regions. This scenario increases the intrinsic probability that new gene copies are located in these regions. In the long term, a recurrence of this situation combined with successive selection events would lead to the progressive accumulation of younger genes in late-replicating regions. The location of new genes in heterochromatin would favor the development of cell type-specific patterns of gene expression. This restriction on gene expression will reduce the selection pressure on new genes, resulting in a weaker impact on the whole organism. In this scenario the rapid development of new traits would contribute to the differential evolution of distinct cell types. Obviously, the influence of other unexplored factors would be expected and should not be ruled out.

The preferential birth of new genes in heterochromatic regions provides a platform that might have facilitated, and that would continue to facilitate, rapid evolution in multicellular species (Fig. 6). In fact, new genes could accumulate mutations faster in late-replicating and heterochromatic regions (Stamatoyannopoulos et al., 2009; Pink and Hurst, 2010), since compact chromatin seems to be prone to suffer DNA damage due to replicative stress (Sulli et al., 2012; Alabert and Groth, 2012). At the same time, it is known that DNA damage promotes heterochromatin formation (Jasencakova and Groth, 2010), such that heterochromatin and replicative stress can be considered as both a cause and consequence of each other. Thus, these processes would constitute a feed-forward loop that can contribute to genetic divergence by fueling the birth of new genes and accelerating their evolution. This scenario, where new genes tend to be born in silenced and mutagenic regions could also help understand the accelerated evolution of young genes reported previously (Albà and Castresana, 2005; Wolf et al., 2009; Vishnoi et al., 2010) in terms of a more relaxed selection pressure and of a higher sequence divergence.

In the light of our results and the scheme proposed, the physical limitations on DNA replication and repair that are imposed by the complexity of certain genomic regions might facilitate rapid evolution in eukaryotic cells. However, the potential influence of structural molecular constraints on the evolution of complexity is only just starting to be understood (Prendergast and Semple, 2011; Fernández and Lynch, 2011; Chambers et al., 2013), and the implications of these structural and mechanistic constraints for evolutionary models must still be investigated in depth. Future assessment of the evolutionary relevance of this proposed global scenario will be necessary, and we anticipate that exploring such issues will further advance our understanding of living systems.

## Materials and Methods

### Ensembl and genomic build versions

We used Ensembl version 61 for all the analyses of the genomic datasets, which corresponds to the human GRCh37.p2 (hg19) and mouse NCBIM37 (mm9) genome builds. We used the Ensembl assembly converter to update the human data in NCBI36 to GRCh37.p2 and the mouse data in NCBIM36 to 37.

### Definition of copy number variable genes

We used accurate gene copy number variation data from a recent study performed on 159 human genomes (including 15 high coverage genomes (Sudmant et al., 2010)). In this study, the authors built genome wide copy number variation (CNV) maps based on a read depth analysis of the corresponding whole-genome shotgun data and they used these maps to estimate the copy number for each individual gene (Sudmant et al., 2010). These authors kindly provided gene copy number estimates for all individuals and 19,315 RefSeq genes. We converted the RefSeq IDs to ENSEMBL IDs using ENSEMBL-Biomart v61 and we retrieved a total of 17,852 ENSEMBL protein-coding genes with copy number data. The genes smaller than 1 Kb were removed as their copy number estimates are unreliable (Sudmant et al., 2010). We focused on autosomal copy-variable genes, including those genes having 4 or more copies, or less than 2 copies, in at least 2 individuals. Based on these criteria, we obtained a set of 1,092 reliable copy-variable autosomal protein-coding genes.

### Phylostratification of gene duplicates

We established an analytical pipeline to perform precise phylostratification (Domazet-Lošo et al., 2007) in a manner similar to that described recently (Roux and Robinson-Rechavi, 2011). We used the gene family phylogenetic reconstructions of ENSEMBL Compara v61 (Flicek et al., 2011) that are based on genes sequenced from 52 different species. ENSEMBL Compara v61 provides 18,583 annotated gene family trees for 876,985 protein coding genes, and it assigns the speciation or duplication events represented by each internal tree node to the phylogenetic level (or age class) where these events are detected (Vilella et al., 2009). We used this information in our pipeline to establish the gene duplication age as that of the phylostratum assigned to the last duplication leading to the birth of the extant protein-coding genes. In order to limit the problems associated to reference genomes of species sequenced with low coverage, we only used the age classes defined by species sequenced with relatively high coverage (at least 5×). Singleton genes were defined as those protein-coding genes without a detectable duplication origin in their gene trees.

According to the aforementioned definition of gene duplication age, the age of a protein-coding duplicated gene (PDG) represents that of the ancestral species in which the duplication event that led to the generation of the extant gene was detected. For this purpose, we only considered duplication events showing a consistency score above 0.3 (Vilella et al., 2009). When this score was exactly 0, we considered that the duplication was an artifact of the phylogenetic reconstruction and we established the gene duplication age in function of the previous node in the tree. Otherwise, we considered the case unclear, such that gene duplication age could not be assigned. Our analysis included the following 14 age classes for human genes: Bilateria, Coelomata, Chordata, Euteleostomi, Tetrapoda, Amniota, Mammalia, Theria, Eutheria (Eutheria + Euarchontoglires), Simiiformes, Catarrhini, Hominidae, HomoPanGorilla and *Homo sapiens* (Fig. 2; Table 1). Although there is increasing evidence in support of the still controversial (Huerta-Cepas et al., 2007; Cannarozzi et al., 2007) Euarchontoglires class (Lunter, 2007; Madsen et al., 2001; Murphy et al., 2001), we decided to remove it and to collapse this into the Eutherian level. This is a conservative option due to the inconsistencies described previously between gene trees and species phylogeny at this level (Huerta-Cepas et al., 2007; Cannarozzi et al., 2007). Given that all non-human primate gene builds in ENSEMBL v61 were annotated by projecting human genes from Ensembl v58, we removed all the human genes in ENSEMBL Compara v61 that were not included in Ensembl v58. The mouse PDGs were grouped in the same age classes as the human PDGs from Bilateria to Eutheria, with the addition of the mouse specific lineage classes: Glires, Rodentia, Murinae and *Mus musculus* (supplementary material Fig. S3; Table 1). Note that only genes duplicated after the Fungal/Metazoan split were classified as PDGs.

### Replication timing in ESCs

We retrieved the probe log-ratios of the processed and normalized replication times for four human ESCs (BG01, BG02, H7 and H9) from the GEO (Barrett et al., 2011) dataset, GSE20027 (Ryba et al., 2010). These log-ratios were ranked separately for each ESC and each probe log-ratio was substituted by its rank. In order to combine the RT profiles in human ESCs into a unique reference system, we assigned each probe its median rank from the four experiments. For each human protein-coding gene, we assigned the median rank that corresponded to the probe closest to the center of the gene. If the closest probe for a gene was found at a distance further than 10 Kb, the gene was no longer considered. All human protein-coding genes were sorted according to these median ranks to estimate the temporal order of replication.

Processed and normalized log-ratios of murine RT correspond to GSE17983 (Hiratani et al., 2010), which contains data for 46C, D3 and TT2 mouse ESCs, were processed in the same manner. The same applies for the RT data from human lymphoblasts (Ryba et al., 2010) and fibroblasts (Yaffe et al., 2010).

### Data processing and statistical analyses

ENSEMBL databases were accessed using the ENSEMBL Perl API Core and Compara (http://www.ensembl.org/info/docs/api/index.html). The data transformations and file parsing needed to run our gene birth dating pipeline were performed using perl (http://www.perl.org). All statistical analyses and plots were carried out using R basic functions (http://cran.r-project.org) and all our code is available upon request.

## Supplementary Material

Supplementary Material
