# Late-replicating CNVs as a source of new genes

**DOI:** 10.1242/bio.20147815

**Published:** 2014-02-27

**Authors:** David Juan, Daniel Rico, Tomas Marques-Bonet, Óscar Fernández-Capetillo, Alfonso Valencia

This Correction relates to *Biol. Open* 2013, 2:1402–1411.

Unfortunately, there were errors in the first published version of this article. These errors are detailed below and the original article has been changed correspondingly.

The affiliation of Oscar Fernández-Capetillo was incorrect. The correct address is Genomic Instability Group, Spanish National Cancer Research Centre (CNIO), Madrid, Spain.

In addition, Fig. 4 was missing the bold text mentioned in the legend. The correct version of the figure is given below.

**Fig. 4.**
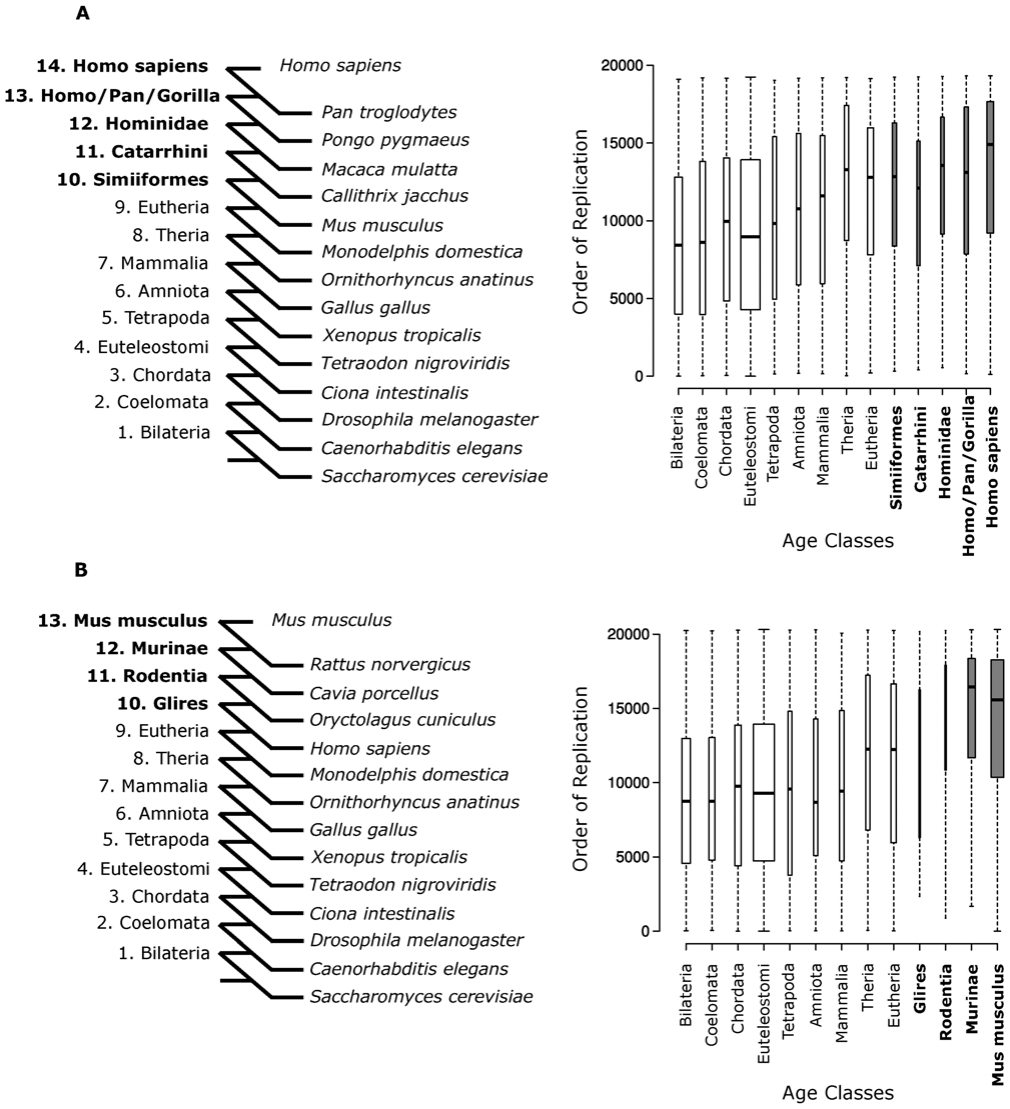
RT mirrors gene duplication phylogeny. (A) RT distribution of human PDGs is correlated with duplication age (rho = 0.21, P-value = 5.1×10^−150^, Spearman's correlation). (B) RT distribution of mouse PDGs is also correlated with duplication age (rho = 0.28, P-value = 5.8×10^−278^). The box width is proportional to the number of PDGs within each figure panel, and the specific human and mouse lineage age classes are indicated in bold. See also supplementary material Figs S2–S4.

We apologise for any confusion caused.

