# Supplementary material for: Late-replicating CNVs as a source of new genes
Source: Biol Open. 2013 Nov 14;2(12):1402–11. doi: 10.1242/bio.20136924 (PMC3863426; doi:10.1242/bio.20136924)
Supplement: Supplementary Material [file supp_2_12_1402__index.html]

Late-replicating CNVs as a source of new genes — Late-replicating CNVs as a source of new genes — Supplementary Material 

# Late-replicating CNVs as a source of new genes

## bio.20136924 Supplementary Material

**Files in this Data Supplement:**

- Supplementary Material - David Juan et al. doi: 10.1242/bio.20136924
