# Supplementary material for: Late-replicating CNVs as a source of new genes
Source: Biol Open. 2013 Nov 14;2(12):1402–11. doi: 10.1242/bio.20136924 (PMC3863426; doi:10.1242/bio.20136924)
Supplement: Supplementary Material [file supp_bio.20136924_bio.20136924-s1.pdf]

Biology Open

Supplementary Material

David Juan et al. doi: 10.1242/bio.20136924

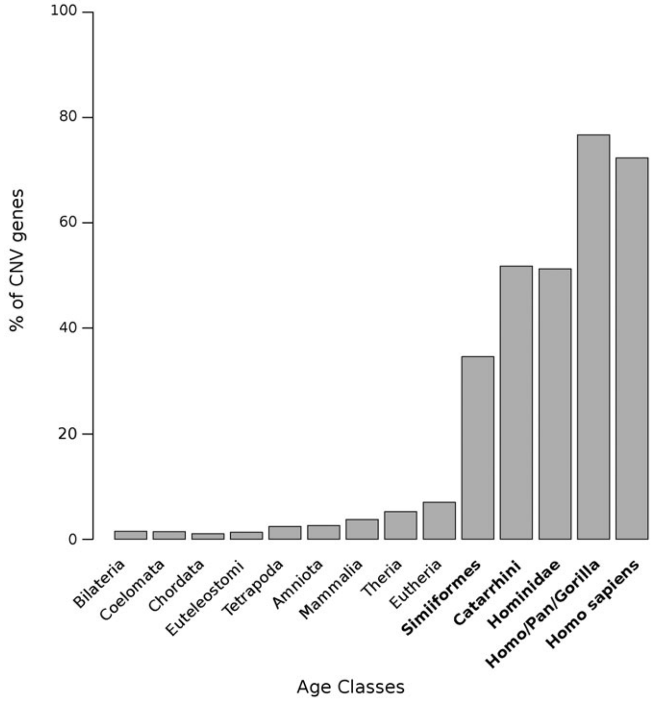

Fig. S1. Proportion of genes affected by CNV-gains in each phylostratum. We repeated the analysis shown in Fig. 2B, excluding CNV losses affecting 163 PDGs and considering only CNV-PDGs affected by gains (929). The proportion of genes affected by CNV gains in each phylostratum is higher in the genes recently duplicated in evolution (P-value $<10^{-150}$, chi-squared test).

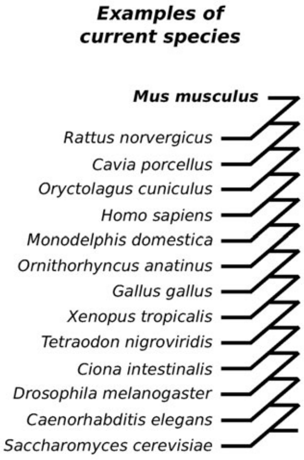

Evolutionary Age classes	Evolutiionary Time Periods (Mya)	Number of duplicates
13. Mus musculus	37-0	2628
12. Murinae	80-37	898
11. Rodentia	81-80	30
10. Glires	90-81	24
9. Eutheria	166-90	472
8. Theria	184-166	287
7. Mammalia	326-184	318
6. Amniota	359-326	275
5. Tetrapoda	420-359	257
4. Euteleostomi	550-420	7090
3. Chordata	570-550	690
2. Coelomata	580-570	564
1. Bilateria	<~580	1144

Fig. S2. Phylostratification of mouse PDGs. The age of a duplicated gene represents the ancestral species in which the duplication event that led to the generation of the extant gene was detected. A total of 14,677 mouse gene duplicates were assigned to one of the 13 different evolutionary age groups (or phylostrata). Representative extant species that define the gene age classes are indicated (see Table 1 for the complete list).

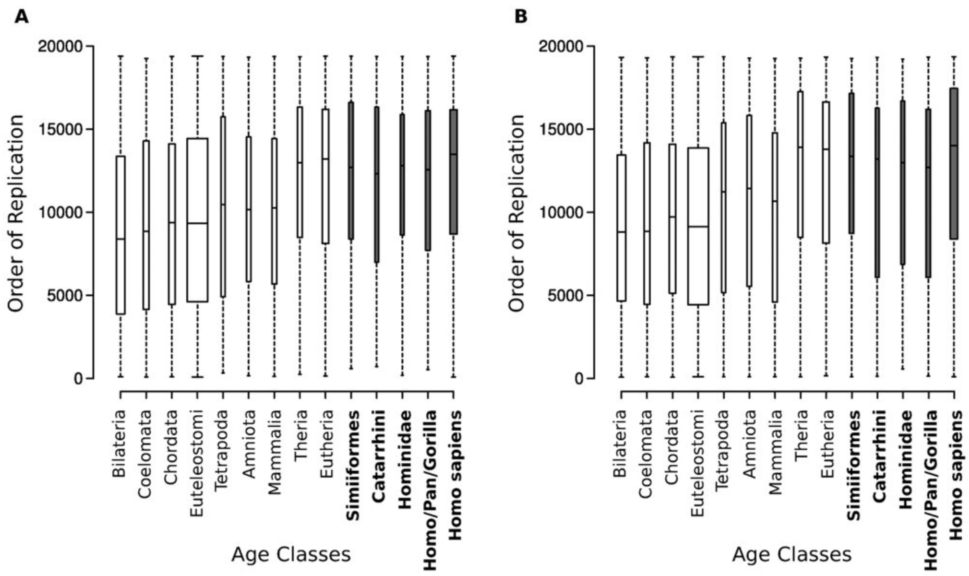

Fig. S3. Replication timing in lymphoblasts and fibroblasts mirrors evolutionary age. Analyses equivalent to Fig. 4A, using alternative replication timing maps from human lymphoblasts and fibroblasts. (A) RT distribution of human PDGs in lymphoblasts (Ryba et al., 2010) is correlated with duplication age (rho=0.18, P-value 2.3×10^{-103}, Spearman's correlation). (B) RT distribution of human PDGs is also correlated with duplication age in fibroblasts (profiles obtained using an alternative methodology; rho=0.17, P-value=1.6×10^{-92}). The box width is proportional to the number of PDGs, and the specific human and mouse lineage age classes are indicated in bold.

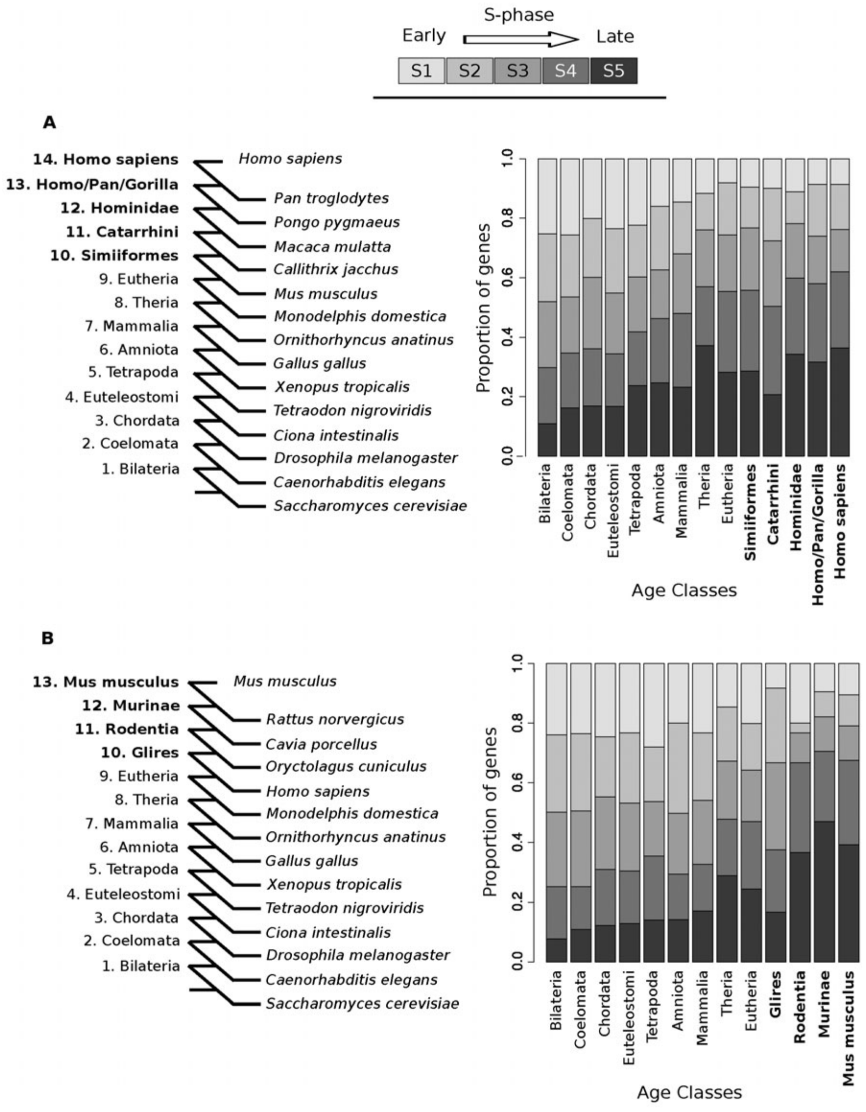

Fig. S4. Replication timing in mouse and human ESCs mirrors evolutionary age: alternative representation. The distribution of genes in distinct RT fractions of human (A) and mouse (B) phylogenies, using extant species to define the gene age classes as indicated (see Table 1 for the complete list). We grouped all genes into five consecutive temporal clusters that contain a similar number of genes (quintiles S1–S5).
